# Paraspinal synovial sarcoma mimicking tuberculosis: A case report and literature review

**DOI:** 10.1097/MD.0000000000041256

**Published:** 2025-01-10

**Authors:** Jie Liu, Xiajie Huang, Xinyun Liang, Xinhua Xian, Yangzhou Mo, Xiaomei Wu, William Lu, Jian Li, Yan Chen

**Affiliations:** a Department of Bone and Joint Surgery, the First Affiliated Hospital of Guangxi Medical University, Nanning, China; b Collaborative Innovation Centre of Regenerative Medicine and Medical BioResource Development and Application Co-constructed by the Province and Ministry, Guangxi Medical University, Nanning, Guangxi, China; c Department of General Medicine, the First Affiliated Hospital of Guangxi Medical University, Nanning, China; d Department of Orthopaedics and Traumatology, the University of Hong Kong, Hong Kong, China; e Department of Spinal Orthopedic Surgery, the First Affiliated Hospital of Guangxi Medical University, Nanning, China.

**Keywords:** case report, paraspinal tumor, spinal tuberculosis, synovial sarcoma

## Abstract

**Rationale:**

Synovial sarcoma (SS) is a rare and highly malignant soft tissue sarcoma. When SS occurs in atypical locations, it can present significant diagnostic challenges. We report a case of paraspinal SS initially misdiagnosed as spinal tuberculosis, highlighting the diagnostic difficulties and the importance of considering SS in the differential diagnosis.

**Patient concerns:**

A 23-year-old woman presented with progressively worsening lower left back pain over 3 weeks, accompanied by weakness and numbness in her left lower limb. She was initially misdiagnosed with spinal tuberculosis at 2 different hospitals based on weakly positive anti-tuberculosis antibodies and imaging findings. Despite ongoing anti-tuberculosis treatment, her condition continued to deteriorate.

**Diagnoses:**

The first surgery revealed findings inconsistent with spinal tuberculosis, but a tumor could not be excluded. However, the initial pathological biopsy was inconclusive. A second surgery confirmed the diagnosis of SS through histopathological examination.

**Interventions:**

The patient underwent a second surgery for mass resection and biopsy confirmation. Unfortunately, by the time the correct diagnosis was made, the disease had metastasized to her lungs, and the optimal window for surgical intervention had been missed.

**Outcomes:**

The patient’s delayed diagnosis resulted in extensive diffuse metastasis to both lungs, significantly impacting her survival.

**Lessons:**

This case underscores the need to consider malignancies such as SS in the differential diagnosis of spinal lesions, particularly when clinical response to treatment is poor. Early diagnosis and timely surgical intervention are critical to improving patient outcomes. Our literature review provides further insights into the characteristics of paraspinal SS and strategies to prevent misdiagnosis, emphasizing the importance of early and accurate diagnosis to enhance patient survival.

## 1. Introduction

Synovial sarcoma (SS) is a rare and highly malignant type of soft tissue sarcoma, accounting for approximately 5% to 10% of all soft tissue sarcoma cases.^[1,2]^ It predominantly affects young adults between the ages of 15 and 40 years and can arise in various anatomical locations, most commonly in the extremities.^[1]^ Paraspinal SS is exceedingly rare and poses diagnostic challenges because of its nonspecific clinical presentation, which may lead to a diagnostic pitfall with the potential for misdiagnosis as more common conditions, such as spinal tuberculosis.^[3]^ Misdiagnosis may lead to inappropriate treatment and incorrect assessment of the prognosis.

The initial symptoms of paraspinal SS can be misleading, often resembling inflammatory or infectious diseases.^[4,5]^ Misdiagnosis can lead to inappropriate treatment and delayed definitive therapy, adversely affecting prognosis. Here, we described a case of a paraspinal SS. Our case emphasizes the diagnostic challenges of paraspinal SS, particularly when the initial presentation is similar to spinal tuberculosis. This case review aims to highlight the characteristics of paraspinal SS, provide insights to prevent misdiagnosis, and stress the importance of early and accurate diagnosis while comprehensively analyzing advancements in treatment.

## 2. Case report

### 2.1. Case presentation

**Figure 1.**
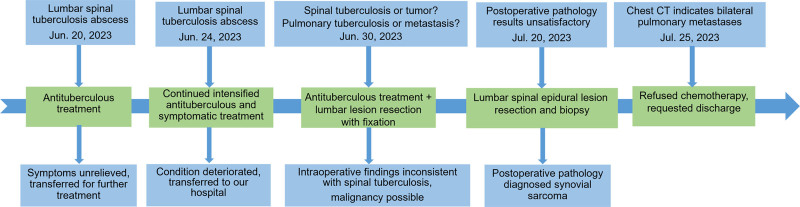
The timeline of the diagnosis and treatment course of the patient in the present case. CT = computed tomography.

A 23-year-old female presented to the Department of Spinal Orthopedic Surgery on June 30, 2023, with a month-long history of lower back pain, which worsened significantly over the preceding ten days, accompanied by progressive numbness and weakness in her lower limbs (Fig. 1). Initially, the pain was intermittent, dull, and localized to the left lower back, not affecting her mobility. Over time, the symptoms escalated, with continuous pain and impaired walking.

The patient was initially diagnosed with a spinal tuberculosis abscess at a local hospital, based on weakly positive anti-tuberculosis antibodies and imaging findings. Despite anti-tuberculosis treatment, her symptoms persisted and worsened. A subsequent lumbar spine magnetic resonance imaging (MRI) revealed a left retroperitoneal mass, supporting the initial diagnosis. However, continued symptomatic and anti-tuberculosis therapy failed to alleviate her condition, prompting her referral to our institution.

### 2.2. Clinical examination and imaging findings

**Figure 2.**
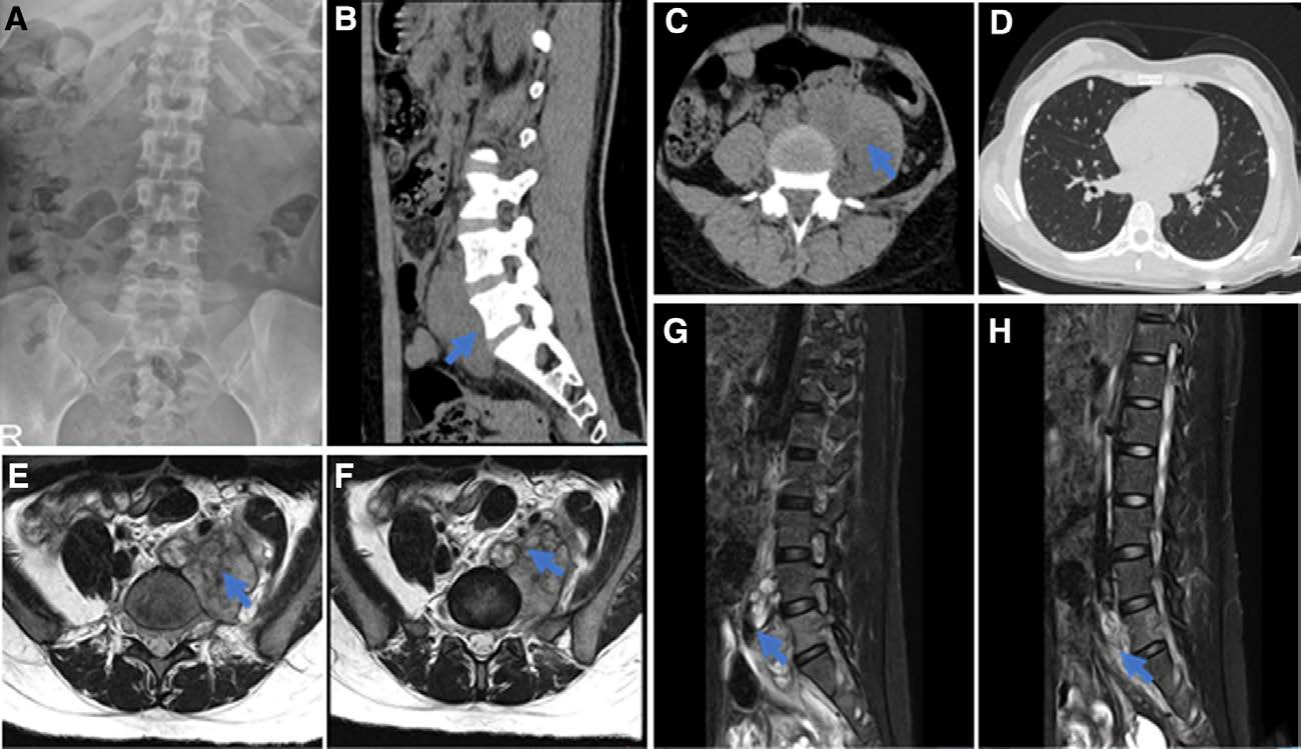
Imaging data before the first surgery. (A) Orthopantomogram of lumbar spine. (B) Sagittal CT image of the lumbar spine. (C) CT transverse section of lumbar spine. (D) Lung CT cross-section. (E, F) lumbar spine MRI cross-sections. (G, H) Lumbar spine MRI sagittal view. CT= computed tomography, MRI = magnetic resonance imaging.

Physical examination revealed significant tenderness and percussion pain over the lumbar vertebra 4 (L4) body surface projection area. Muscle strength was graded 3/5 in the left lower limb and 4/5 in the right, with a positive right straight leg raising test but no pathological reflexes. Initial imaging at our hospital identified a left lumbar psoas major swelling from L4 to sacral vertebra 1 (S1) and an irregular soft tissue mass posterior to the L4-S1 vertebrae with internal signal heterogeneity (Fig. 2A–H). Computed tomography (CT) scans of the chest revealed diffuse pulmonary nodules of varying sizes, raising suspicion for metastatic disease or disseminated tuberculosis (Fig. 2D).

### 2.3. Phase I surgery and postoperative course

**Figure 3.**
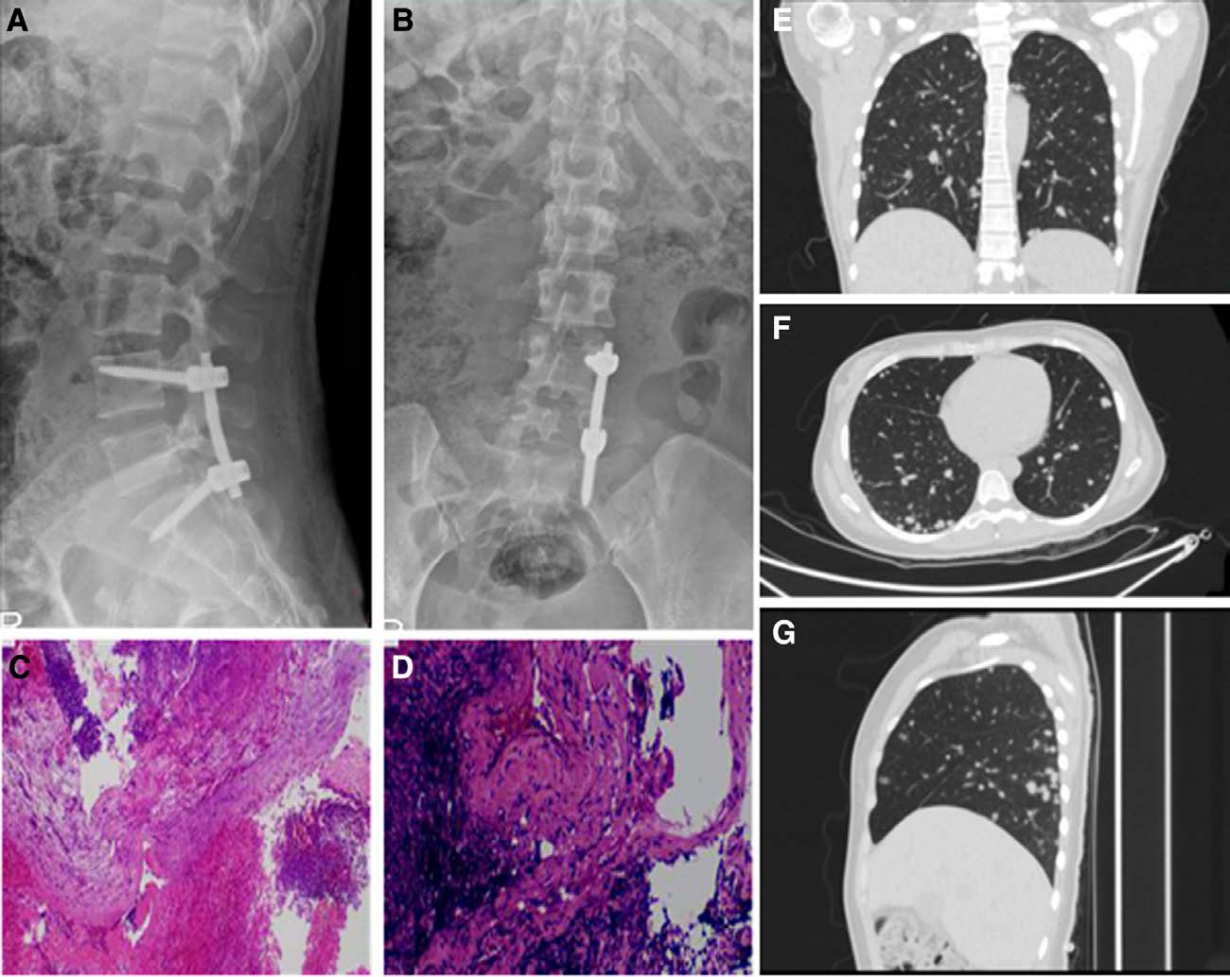
Imaging data after the first surgery. (A, B) Review of lumbar spine front and side view pictures. (C, D) Hematoxylin and eosin stained pictures of the first pathological examination. (E–G) Postoperative review chest computed tomography pictures.

Given her neurological symptoms and imaging findings, the patient underwent posterior lumbar internal fixation, nerve decompression, and biopsy on July 3, 2023 (Fig. 3A–D). Intraoperative findings revealed grayish-white fish-like tissue, which was sent for pathological examination. Postsurgical CT scans showed diffuse pulmonary nodules, which had grown compared to previous scans (Fig. 3E–G). Despite sputum and wound secretion cultures being negative for Mycobacterium tuberculosis, respiratory specialists favored metastatic lung tumors over tuberculosis based on imaging findings.

### 2.4. Phase II surgery and definitive diagnosis

**Figure 4.**
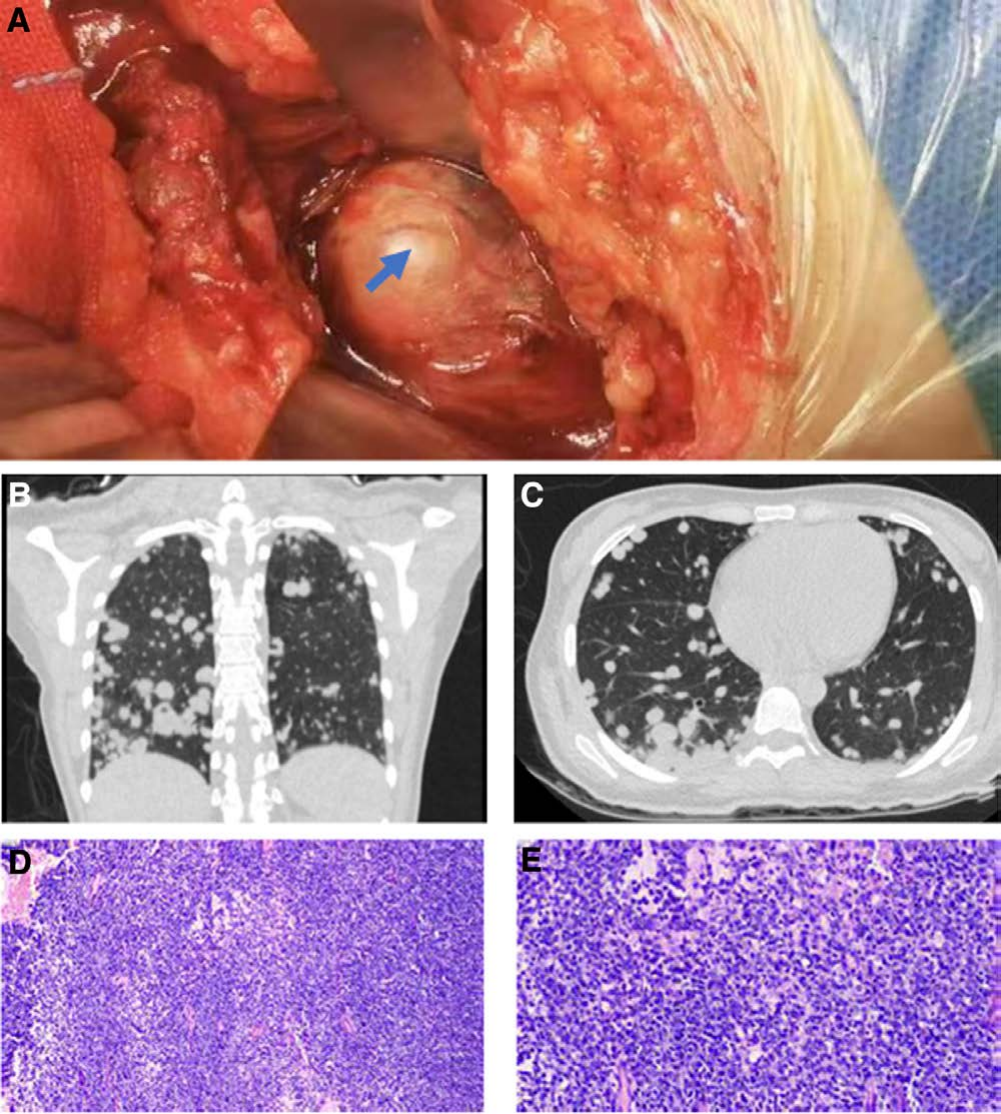
Intraoperative and postoperative images of the second surgery. (A) intraoperative photographs of the second surgery. (B, C) Postoperative chest computed tomography pictures. (D, E) Hematoxylin and eosin stained pictures of the second pathological examination.

Due to inconclusive pathology and persistent symptoms, a second surgery was performed on July 20, 2023, through a left anterior abdominal approach to excise the lumbar psoas major tumor (Fig. 4A). Pathology confirmed a mesenchymal-origin round short spindle cell tumor with immunohistochemical results consistent with SS. Key markers included Bcl-2 (+), ALK (+), CD99 (+), and Ki-67 (~80%+) (Fig. 4D and E).

### 2.5. Outcome and follow-up

Postoperative imaging on July 25, 2023, revealed further progression of pulmonary nodules (Fig. 4B and C). Despite oncology recommendations for chemotherapy, the patient declined treatment and opted for discharge. Upon discharge, her general condition was stable, and she was advised to follow up for palliative care.

## 3. Discussion

To review the characteristics of paraspinal SS and explore factors affecting diagnosis and treatment decisions, we conducted a thorough case review. The literature review utilized 2 major databases, PubMed (https://pubmed.ncbi.nlm.nih.gov/) and Web of Science (https://www.webofscience.com/wos/woscc/basic-search), searching for relevant English-language articles from 2004 to 2024. The search keywords were “spine,” “paraspinal,” “synovial sarcoma,” and “case report.” A total of 59 articles related to paraspinal SS and the full texts of these articles were available in English. These 28 cases were arranged chronologically and summarized (Table 1).^[4–31]^

**Table 1:** Clinical data of 28 cases with paraspinal synovial sarcoma.

Authors, years	Age (yr)/sex	Symptoms and signs	Radiology findings	Misdiagnosis/metastasis	Biphasic (BP) or monophasic (MP)/location	Treatments	Outcomes/follow-up (mo)
Wang et al,^[6]^ 2022	16/F	Upper back pain, paraparesis, hypalgesia	CT: compression fracture and right-sided soft tissue mass MRI: evident patchy calcification at the edge of the vertebral body and spinal cord compression	NO/NO	MP/T7–T8	Surgery, radiotherapy, and chemotherapy	Alive/8
Zhang et al,^[7]^ 2021	13/F	Pain in the left lower limb	CT: large mixed-density tumor in the left abdominal cavity at the thoracic level and extensive calcifications in the spinal canal and paraspinal region	NO/NO	MP/T11–L4	Surgery and chemotherapy	Alive/4
Feng et al,^[8]^ 2020	56/F	Low back pain	CT: lungs: Multiple pulmonary nodules L2: bony erosion of L2 vertebra and spinal canal stenosis	NO/NO	MP/L2	Surgery and radiotherapy	Alive/NA
Zimelewicz Oberman et al,^[9]^ 2020	62/M	Progressive thoracic pain, gait weakness, sudden paraplegia, and urinary/bowel dysfunction	CT: thoracic paravertebral mass and cord compression MRI: space-occupying lesion, osteolytic lesions	NO/NO	BP/T5–T9	Radiotherapy and chemotherapy	NA/NA
Alshehri et al,^[10]^ 2020	12/F	Lower-mid back pain extending to the left anterior thigh	CT: left paraspinal soft tissue mass from T12 to L4 level	NO/NO	MP/T12–L4	Surgery, radiotherapy, and chemotherapy	Alive/12
Subramanian et al,^[11]^ 2020	46/F	Midback pain and bilateral lower limb weakness	PET-CT, MRI: dumbbell-shaped thoracic spinal cord tumor at T7–T8 level with erosion of lamina and pedicle	Nerve sheath tumor/NO	MP/T7–T8	Surgery and radiation	Alive/12
Najib et al,^[12]^ 2018	44/M	Chronic lower back pain	CT: spine: a sclerotic and lytic lesion Lungs: multiple bilateral pulmonary nodules along with mediastinal and hilar lymphadenopathy	NO/NO	MP/T12	NA	NA/NA
Shah et al,^[4]^ 2018	40/M	Shoulder pain, progressive quadriparesis, back bulge, and urinary incontinence	CT: large intradural-extramedullary mass with neural compression and paraspinal extension from C5 to T5	Spinal tuberculosis/lung metastases	MP/C5–T5	Surgery and radiation	Alive/24
Guo et al,^[5]^ 2016	10/M	Progressive back pain, low-grade fever, and acute paraplegia	MRI: extradural gadolinium-enhancing lesion at T9–10	Inflammatory abscess/NO	NA/T9–T10	Surgery and radiation	Alive/6
Yang et al,^[13]^ 2016	20/M	Hypoesthesia in his left limbs	MRI: intramedullary mass with peritumoral edema at C2	NO/NO	MP/C2	Surgery	Died/1
Chen et al,^[14]^ 2016	20/F	Low back pain, left low back swelling, and paraparesis	CT: left paraspinal mass with bone erosion, and T12–L2 spinal cord compression MRI: paraspinal mass with heterogeneous cystic and solid features	NO/NO	MP/T12–L2	Surgery and radiation	NA/NA
Cao et al,^[15]^ 2014	26/M	Low back pain	CT: spine: T7 bony erosion, and no soft tissue mass Lungs: multiple nodules with clear boundaries MRI: tumor in the canal and cord compression	NO/NO	BP/T7	Surgery, radiotherapy, and chemotherapy	Alive/12
Kim et al,^[16]^ 2014	29/M	Neck lump with right arm pain and limited movement	CT: lobulated calcified paravertebral space mass at the right occipitocervical junction	NO/NO	BP/C2-C3	Surgery and radiation	Alive/24
Peia et al,^[17]^ 2013	7/F	Progressive anterolateral knee pain with gait disturbances	MRI: oval, enhancing lesion at L4–L5 and left neural foramen widening	Schwannoma/NO	BP/L4–L5	Surgery and chemotherapy	Alive/60
Kim et al,^[18]^ 2013	17/M	Diving-related posterior neck pain and bilateral upper limb numbness	X-ray: C3 vertebra expansile osteolytic lesion with cortical thinning MRI: vertebral epidural mass in C3 with extraosseous extension and enhancement	NO/NO	MP/C2–C3	Surgery and chemotherapy	NA/NA
Yonezawa et al,^[19]^ 2012	11/F	Low back pain	MRI: an intradural, extramedullary, and uniformly enhancing mass that extended from L3 to L4	NO/NO	MP/L3–L4	Surgery and radiation	Alive/60
Naphade et al,^[20]^ 2011	14/M	Shoulder pain and right limb weakness	MRI: extramedullary oval lobulated mass lesion in C6–C7 intervertebral foramen with nerve root compression	NO/NO	MP/C6–C7	Surgery	Alive/60
Zairi et al,^[21]^ 2011	36/M	Neck mass with pain	CT: left lung nonspecific node MRI: posterior cervical soft tissue tumor	NO/lung metastases	BP/C1–C2	Surgery and radiation	Died/72
Foreman et al,^[22]^ 2011	29/M	Posterior cervical spine muscle discomfort/pain after weight lifting	CT: cystic mass at C4-C5, nonenhancing MRI: septated kidney bean-shaped mass with high signal	NO/NO	BP/C3–T2	Surgery, radiotherapy and chemotherapy	Alive/72
Liu et al,^[23]^ 2010	12/M	Lameness, intermittent bilateral leg pain, and voiding dysfunction	CT: S3–C1 vertebral involvement with tumor calcification MRI: large enhancing sacral lesion below S2	NO/chest	BP/S3–S2	Surgery and radiotherapy	Died/21
Koehler et al,^[24]^ 2009	60/M	Abdominal pain with radiation to back, dyspnea on exertion, increased pain on standing	CT: right paraspinal mass, T9 vertebral lysis MRI: large right-sided paraspinal mass at eighth and ninth ribs	NO/NO	MP/T7–T10	Surgery and radiotherapy	Alive/9
Barus et al,^[25]^ 2009	14/F	Chronic lumbar pain, neurologic symptoms, and a palpable mass	CT: soft tissue mass, lumbar spine, local bone erosion MRI: lumbar spine, spinal canal, epidural involvement, neural compression	NO/NO	MP/L2–L5	Surgery, radiotherapy, and chemotherapy	Alive/69
Ravnik et al,^[26]^ 2009	32/M	Rapidly progressing paraparesis	MRI: intramedullary epidural mass	NO/NO	MP/T12–L1	Surgery, radiotherapy, and chemotherapy	Died/12
Mullah-Ali et al,^[27]^ 2008	14/F	Intermittent knee pain post-fall, pelvic tilt, persistent night back pain, and left leg weakness	CT: left paraspinal mass, narrowing of the L3–L4 neural exit foramen and spinal canal MRI: multilobulated heterogeneous mass at the L3–L4 level	NO/pulmonary metastases	MP/L3–L4	Surgery, radiotherapy, and chemotherapy	Alive/6
De Ribaupierre et al,^[28]^ 2007	11/F	Cervicobrachialgia and weakness in the right arm	MRI: enhancing heterogeneous intradural mass at C6–C7	NO/NO	MP/C6–C7	Surgery, radiotherapy, and chemotherapy	Died/60
Greene et al,^[29]^ 2007	11/F	Back pain	MRI: intradural masses at C6, T2, T5, T8, and L1 levels; additional nodules of enhancement at L2–L4	NO/leptomeningeal metastasis	MP/L2–L4	Surgery, radiotherapy, and chemotherapy	Died/14
Sakellaridis et al,^[30]^ 2006	36/F	Low back pain, walking difficulties, and urinary incontinence	MRI: recurrent epidural mass at the L2–L3 level	NO/brain, lungs, and spinal metastases	MP/L2–L3	Surgery and radiotherapy	Died/18
Suh et al,^[31]^ 2005	44/M	Right-sided sciatica	MRI: right epidural, paravertebral mass, widened L4–L5 neural foramen, eroded L5 articular process	NO/NO	BP/L4–L5	Surgery and radiotherapy	Alive/5

BP = biphasic, C = cervical, CT = computed tomography, F = female, L = lumbar, M = male, MP = monophasic, MRI = magnetic resonance imaging, NA = not available, PET-CT = positron emission tomography-computed tomography, T = thoracic.

In all the 28 reported cases, the average patient age was 26.5 years, with a standard deviation of 16.53 years, indicating that paraspinal SS predominantly affects young individuals. The age range was broad, spanning from 7 to 62 years, showing that this tumor can impact almost any age group. Males were slightly more affected than females, but the gender difference was not particularly significant.

The clinical features of paraspinal SS present a range of symptoms closely related to the location and size of the tumor. Patients often first experience localized pain in the tumor area, which may progressively worsen over time, prompting them to seek medical attention. As the tumor grows and compresses surrounding structures, patients may develop neurological deficits such as limb weakness, numbness, sensory reduction or abnormalities, and even gait disturbances, affecting daily walking. These symptoms are typically associated with nerve root compression. In some cases, prolonged neurological impairment can lead to muscle atrophy, especially in the affected limb. Additionally, bladder and bowel dysfunction may occur if the tumor compresses the lower spinal cord. Severe spinal cord compression can result in sudden paralysis. Rare symptoms like fever may also occur, overlapping with infectious diseases and necessitating comprehensive evaluation based on the patient’s overall clinical presentation.

Imaging characteristics of paraspinal SS typically include a tumor in the epidural space of the spinal cord, which may appear as a paraspinal soft tissue mass on CT scans, often showing mixed density with shapes such as patchy, dumbbell-shaped, multilobulated, or oval. Vertebral compression fractures, bone destruction, and erosion of the vertebral body may be observed, sometimes with patchy calcifications along the vertebral margins. The tumor may compress the spinal cord or nerve roots, causing deformation or displacement. On MRI, SS shows high signal intensity on T1-weighted images and intermediate signal intensity on T2-weighted images, possibly due to hemorrhage. SS can exhibit a “triple signal intensity” pattern: high, intermediate, and low signals. Postcontrast MRI typically shows heterogeneous enhancement. Tumors often erode the vertebral bones, leading to cortical disruption. Additionally, the tumor may compress adjacent blood vessels and neural structures, causing displacement or signal changes. In advanced cases, distant metastases, such as to the lungs or other bones, may occur, with the lungs being the most common site. Tissue biopsy is considered the gold standard for determining the nature of the tumor. SS is a diverse malignant tumor with varying subtypes and biological behaviors. Histologically, the tumor can be biphasic or monophasic, with monophasic cases (20/28) being more common than biphasic cases (8/28) in paraspinal SS. Late-stage paraspinal SS metastasis to other sites significantly impacts patient prognosis. The most common metastatic site is the lungs, followed by intravertebral metastasis. Although rare, leptomeningeal metastasis can occur in paraspinal SS patients, leading to a poor prognosis and shorter survival time.

Comprehensive treatment of paraspinal SS primarily involves radical surgery supplemented with local radiotherapy and chemotherapy. In our case review, the majority of patients (26/28) underwent surgical treatment. Surgical resection is the primary treatment choice for SS, especially when complete or near-complete tumor removal can be achieved, leading to better short-term outcomes. However, not all cases can achieve complete tumor resection, and there is a high risk of recurrence. In such cases, radiotherapy is an important adjunctive treatment, reducing the risk of local recurrence or treating tumors that are not accessible surgically. When metastasis occurs, chemotherapy plays a crucial role in SS treatment, with cyclophosphamide and adriamycin being the first choices.

SS is a rare and complex disease that is prone to misdiagnosis due to its symptoms and imaging characteristics overlapping with many other conditions. In our review, we found that it was often misdiagnosed as a nerve sheath tumor, spinal tuberculosis, inflammatory abscess, or schwannoma.^[4,5,11,17]^ These conditions can cause spinal cord compression, localized pain, and neurological deficits, which overlap with the manifestations of paraspinal SS, increasing the difficulty of diagnosis. Improving the first diagnosis rate and surgical intervention in the early stages of tumor development is often a more ideal and effective treatment method. As shown in this case, the patient’s tumor was initially mistaken for a paraspinal cold abscess caused by spinal tuberculosis and was treated with an anti-tuberculosis regimen for 1 month at an outside hospital. During this period, the patient missed the optimal treatment window and developed extensive lung metastasis.

We analyzed the factors that contributed to the misdiagnosis of this patient as having lumbar tuberculosis with a paraspinal tuberculosis abscess. The weakly positive anti-tuberculosis antibody result, combined with MRI findings that initially suggested spinal tuberculosis, contributed to this misdiagnosis. The MRI showed involvement of the L4-S1 intervertebral disc, which, along with the patient’s history of irregular fever, raised suspicion for lumbar tuberculosis. However, it is important to note that a weakly positive anti-tuberculosis antibody does not necessarily indicate active tuberculosis. It could either be a false positive or reflect residual antibodies from a past tuberculosis infection that had resolved.

The issue was not a lack of key details in the imaging findings for SS but rather the overlap in imaging features between SS and tuberculosis. MRI findings indicated a mass in the left lumbar psoas muscle with clear boundaries, uneven signal intensity, and outward displacement of the muscle. Furthermore, a biopsy was not performed initially, which delayed diagnosis. We hypothesize that the patient’s use of anti-tuberculosis medications early in the course of treatment may have been a factor in this delay, as it was thought to prevent further development of the tuberculosis abscess. Moreover, there were concerns that performing a biopsy while the infection was not under control might lead to undesirable consequences, such as the spread of infection. Ultimately, the failure to perform a biopsy in the early stages hindered timely diagnosis, highlighting the importance of considering SS in the differential diagnosis and the need for prompt tissue diagnosis to clarify pathological findings.

Our patient experienced rapid, extensive lung metastasis due to early misdiagnosis, missing the optimal time for surgical treatment. We can predict a short survival time for such cases. We hope that by sharing this case, we can draw attention to this type of malignant tumor with relatively low incidence so that you can be prepared to make differential diagnoses, shorten the diagnostic period, and provide early surgical and pharmacological interventions to maximize the survival time and quality of life for your patients when encountering such cases.

## 4. Conclusion

This case highlights the importance of including SS in the differential diagnosis of spinal lesions. Early diagnosis and timely intervention are essential to improving patient outcomes.

## Author contributions

**Writing – original draft:** Jie Liu, Xiajie Huang, Xinyun Liang.

**Writing – review & editing:** Jie Liu, Xiajie Huang, Jian Li, Yan Chen.

**Formal analysis:** Xinyun Liang, Yangzhou Mo.

**Validation:** Xinyun Liang.

**Investigation:** Xinhua Xian.

**Software:** Xiaomei Wu.

**Supervision:** William Lu, Yan Chen.

**Conceptualization:** Jian Li.
